# Characterization of tenascin-C as a novel biomarker for asthma: utility of tenascin-C in combination with periostin or immunoglobulin E

**DOI:** 10.1186/s13223-018-0300-7

**Published:** 2018-11-19

**Authors:** Mina Yasuda, Norihiro Harada, Sonoko Harada, Ayako Ishimori, Yoko Katsura, Yukinari Itoigawa, Kei Matsuno, Fumihiko Makino, Jun Ito, Junya Ono, Kazunori Tobino, Hisaya Akiba, Ryo Atsuta, Kenji Izuhara, Kazuhisa Takahashi

**Affiliations:** 1 Department of Respiratory Medicine, Juntendo University Faculty of Medicine and Graduate School of Medicine, 3-1-3 Hongo, Bunkyo-ku, Tokyo, 113-8431 Japan; 2 Department of Respiratory Medicine, Iizuka Hospital, Fukuoka, Japan; 3 Research Institute for Diseases of Old Ages, Juntendo University Faculty of Medicine and Graduate School of Medicine, Tokyo, Japan; 4 Atopy (Allergy) Research Center, Juntendo University Faculty of Medicine and Graduate School of Medicine, Tokyo, Japan; 5 Shino-Test Corporation, Sagamihara, Japan; 6 Department of Immunology, Juntendo University Faculty of Medicine and Graduate School of Medicine, Tokyo, Japan; 7 Division of Medical Biochemistry, Department of Biomolecular Sciences, Saga Medical School, Saga, Japan

**Keywords:** Tenascin-C, Periostin, Asthma, Type 2 biomarker, Immunoglobulin E

## Abstract

**Background:**

Extracellular matrix proteins tenascin-C (TNC) and periostin, which were identified as T-helper cell type 2 cytokine-induced genes in human bronchial epithelial cells, accumulate in the airway basement membrane of asthmatic patients. Although serum periostin has been accepted as a type 2 biomarker, serum TNC has not been evaluated as a systemic biomarker in asthma. Therefore, the objective of this study was to evaluate whether serum TNC can serve as a novel biomarker for asthma.

**Methods:**

We evaluated 126 adult patients with mild to severe asthma. Serum TNC, periostin, and total IgE concentrations were quantified using enzyme-linked immunosorbent assays.

**Results:**

Serum TNC levels were significantly higher in patients with severe asthma and high serum total IgE levels. Patients with both high serum TNC (> 37.16 ng/mL) and high serum periostin (> 95 ng/mL) levels (n = 20) or patients with both high serum TNC and high serum total IgE (> 100 IU/mL) levels (n = 36) presented higher disease severity and more severe airflow limitation than patients in other subpopulations.

**Conclusions:**

To our knowledge, this is the first study to show that serum TNC levels in asthmatic patients are associated with clinical features of asthma and that the combination of serum TNC and periostin levels or combination of serum TNC and total IgE levels were more useful for asthma than each single marker, suggesting that serum TNC can serve as a novel biomarker for asthma.

**Electronic supplementary material:**

The online version of this article (10.1186/s13223-018-0300-7) contains supplementary material, which is available to authorized users.

## Background

Although the mechanisms of heterogeneous chronic inflammatory disorders of the airway, including bronchial asthma, are not fully clarified, airway inflammation and remodeling typically occur in these pathologies [1–3]. Asthma is characterized by inflammation of the airways associated with excessive deposition of the extracellular matrix, including basement membrane thickening, mucous cell metaplasia, epithelial shedding, angiogenesis, inflammatory cell infiltration, and smooth muscle cell and lung fibroblast proliferation [4]. An increase in the number of lung fibroblasts characterized by collagen synthesis and in both tenascin and periostin deposition within the basement membrane matrix may occur in response to allergen challenge in asthmatic patients [5, 6].

Although a variety of cell types are involved in allergic airway inflammation, antigen-specific CD4^+^ T-helper cell type 2 (Th2) and type 2 innate lymphoid cells, which secrete Th2 cytokines such as interleukin (IL)-4 and IL-13, are believed to drive asthma pathobiology [7, 8]. Previous microarray analyses identified tenascin-C (TNC) and periostin as IL-4- or IL-13-induced genes in human bronchial epithelial cells [9–13]. Both TNC and periostin are glycoproteins that are secreted into the extracellular matrix. Previous studies suggested that periostin may promote eosinophil infiltration into the asthmatic airway during inflammation and serum periostin may be a systemic biomarker for eosinophilic airway inflammation and disease severity in asthmatic patients [6, 14–18]. It has also been reported that serum periostin has the potential as a prognostic biomarker to predict the risk of a decline in forced expiratory volume in 1 s (FEV_1_) in late-onset and eosinophil-dominant asthmatic patients [19–21].

TNC is prototypic of the TN family and supports the migration of inflammatory cells from the interstitium to the airspace. TNC is highly expressed in human lung during embryonic development, and its expression is especially strong in the extracellular matrix underlying the airway epithelium during the gestational stages [22, 23]. Although TNC expression is less abundant and more restricted in normal adult tissues, TNC expression in the airway subepithelial reticular basement membrane in asthmatic patients is prominently increased after allergen challenge and is a histopathological subepithelial marker to detect disease activity in asthma [24–26]. The thickness of TNC deposition was correlated with the number of eosinophils, T-lymphocytes, and IL-4-positive cells in bronchial mucosa of atopic asthmatics [27]. Previous studies using TNC-deficient mice suggested that TNC provides protection against ovalbumin-induced Th2-driven airway inflammation [28]. Moreover, treating asthmatics with mepolizumab, an anti-IL-5 monoclonal antibody for severe asthma, significantly decreased airway eosinophil numbers and significantly reduced TNC deposition in the airway subepithelial reticular basement membrane when compared with placebo [25]. Furthermore, one report has been demonstrated that serum TNC levels were significantly higher in patients with refractory asthma than in non-refractory asthma and normal volunteers [29]. Although these reports indicated that TNC in asthmatic patients may play a key role in Th2/type 2 airway inflammation, serum TNC has not been evaluated as a potential biomarker of Th2/type 2 airway inflammation and asthma. Therefore, in the present study, we evaluate whether serum TNC levels can serve as a novel biomarker for asthma.

## Methods

### Patients

Consecutive patients with mild to severe asthma, who were aged 20 years or older, were recruited with informed consent from our outpatient clinic at Juntendo University Hospital (Tokyo, Japan). Asthma was diagnosed by a clinical history of episodic symptoms with airflow limitation and by either variation in pulmonary function monitored by forced expiratory volume in 1 s (FEV_1_) or peak expiratory flow (PEF) in accordance with the Global Initiative for Asthma (GINA) guidelines [30]. The disease severity was also assessed in accordance with the GINA guidelines [30]. The present study was reviewed and approved by the Juntendo University Research Ethics Committee (Tokyo, Japan). Written informed consent was obtained from each patient before their participation in the study. This study was registered in the UMIN Clinical Trial Registry (UMIN000009968) on February 5, 2013 (http://www.umin.ac.jp/). Patients having any of the following criteria were excluded: a diagnosis of chronic obstructive pulmonary disease defined by the Global Initiative for Chronic Obstructive Lung Disease guidelines [31] and any current respiratory disorder other than asthma.

The asthma control test (ACT) score, pulmonary function parameters, and fractional exhaled nitric oxide (FeNO) levels were measured. FeNO levels were measured in accordance with the American Thoracic Society recommendations at a constant flow of 0.05 L/s against an expiratory resistance of 20 cm water with a chemiluminescence analyzer (NOA 280i; Sievers, Boulder, CO, USA). On the same day these clinical examination and venous blood sampling were performed.

### Quantification of serum periostin and TNC levels

The sera of patients were collected after density-gradient centrifugation of blood samples and frozen at − 80 °C. Periostin levels were measured with an enzyme-linked immunosorbent assay (ELISA) (Shino test, Sagamihara, Japan), as described previously [32]. TNC was simultaneously quantified in thawed serum using the human TNC ELISA kit (IBL Co. Ltd, Gunma, Japan) [33, 34].

### Statistical analysis

Sample normality was examined using the D’Agostino–Pearson test. Differences in parameters between populations were analyzed for significance using Student’s *t* test, the Mann–Whitney *U* test, the Chi square test, and Fisher’s exact test as appropriate. For correlation between variables, the Pearson’s correlation coefficient and Spearman’s rank correlation coefficient, which is denoted as r_s_ for a sample statistic, were used where appropriate. One-way ANOVA followed by the Tukey test and Kruskal–Wallis test followed by the Dunn test were used for multigroup analysis. Differences were statistically significant when *P* values were 0.05 or less. Statistical analyses were performed using Graph Pad Prism version 6 software (GraphPad Software, Inc., La Jolla, CA, USA). A Th2-high subgroup was defined as both a serum total immunoglobulin E (IgE) level of > 100 IU/mL and a peripheral blood eosinophil count of ≥ 0.14 × 10^9^ cells/L [13, 35, 36].

## Results

### Baseline characteristics

We first determined the baseline characteristics of asthmatic patients (Table 1). This study enrolled 126 patients with mild to severe asthma, including 13 (10.3%) in GINA treatment steps 1 and 2, 32 (25.4%) in step 3, 57 (45.2%) in step 4, and 24 (19.0%) in step 5. The male to female ratio was 43:83, and the median age was 53 years (range 20–86 years). The mean (± standard deviation) duration of asthma was 18.83 ± 15.95 years, and the mean FEV_1_/forced vital capacity (FVC) ratio was 73.46 ± 10.3% (Table 1). We also compared the characteristics of 45 patients (35.7%) included in GINA treatment steps 1–3 (GINA step 1–3 group) and 81 patients (64.3%) included in GINA treatment steps 4 and 5 (GINA step 4 + 5 group) (Table 1). In the GINA step 4 + 5 group, the male to female ratio (*P* = 0.032), smoking history (in pack-years) (*P* = 0.003), ACT score (*P* < 0.001), FVC (*P* = 0.004), percent predicted FVC (%FVC) (*P* = 0.013), PEF (*P* = 0.037), FeNO levels (*P* = 0.037), and serum periostin concentrations (*P* = 0.012) were significantly lower than those in the GINA step 1–3 group. Conversely, the never-smoker/current and ex-smoker ratio which was performed by Fisher’s exact test (*P* = 0.002, data not shown), daily dose of inhaled and oral corticosteroids (*P* < 0.001 and *P* = 0.014, respectively), and serum TNC concentrations (*P* = 0.002) were significantly higher in the GINA step 4 + 5 group compared with the GINA step 1–3 group (Table 1).

**Table 1:** Baseline characteristics of the study population

	Total	GINA step 1–3	GINA step 4 + 5	*P* value
	n = 126	n = 45	n = 81	
Sex (M/F), n (%)	43 (34.1)/83 (65.9)	21 (46.7)/24 (53.3)	22 (27.2)/59 (72.8)	0.032*
Age (years)	53.91 ± 15.86	55.47 ± 15.66	53.05 ± 16.01	0.415
Age at asthma onset (years)	35.08 ± 22.05	36.87 ± 22.46	34.09 ± 21.90	0.437
Duration of asthma (years)	18.83 ± 15.95	18.60 ± 17.20	18.96 ± 15.32	0.613
BMI (kg/m^2^)	24.00 ± 4.88	23.30 ± 4.08	24.38 ± 5.26	0.317
Smoking history (never/ex/current), n (%)	79 (62.7)/42 (33.3)/5 (4.0)	20 (44.4)/23 (51.1)/2 (4.4)	59 (72.8)/19 (23.4)/3 (3.7)	0.006*
Pack year smoking history (pack year)	5.63 ± 10.79	9.00 ± 13.74	3.75 ± 8.25	0.003*
Atopic predisposition, n (%)	99 (78.6)	33 (73.3)	66 (81.5)	0.365
AERD, n (%)	12 (9.5)	1 (2.2)	11 (13.6)	0.055
Atopic dermatitis, n (%)	27 (21.4)	10 (22.2)	17 (21.0)	1.000
Allergic rhinitis, n (%)	66 (52.4)	24 (53.3)	42 (51.9)	1.000
Chronic sinusitis, n (%)	38 (30.2)	13 (28.9)	25 (30.9)	0.843
Daily dose of ICS (FP equivalent dose, µg)	584.13 ± 383.49	177.78 ± 92.05	809.88 ± 283.99	< 0.001*
Daily dose of OCS (PSL equivalent dose, mg)	0.35 ± 1.41	0.00 ± 0.00	0.55 ± 1.73	0.014*
ACT score, n = 125	23.20 ± 2.82	24.29 ± 1.47	22.59 ± 3.20	< 0.001*
FeNO (ppb)	55.04 ± 43.69	65.31 ± 53.19	49.34 ± 36.53	0.037*
Peripheral neutrophils (cells/μL)	4022.36 ± 1492.54	3696.30 ± 1115.92	4203.51 ± 1644.02	0.113
Peripheral eosinophils (cells/μL)	263.54 ± 236.05	231.11 ± 180.79	281.55 ± 261.08	0.775
Serum IgE (IU/mL)	616.37 ± 1686.39	467.19 ± 718.39	699.24 ± 2034.77	0.731
Th2-high^†^, n (%)	53 (42.1)	21 (46.7)	32 (39.5)	0.457
Serum periostin (ng/mL)	87.65 ± 34.49	94.62 ± 30.83	83.78 ± 35.96	0.012*
Serum TNC (ng/mL)	39.49 ± 25.18	30.95 ± 16.69	44.23 ± 27.82	0.002*
FVC (L)	3.22 ± 0.95	3.51 ± 0.87	3.06 ± 0.96	0.004*
%FVC (predicted, %)	103.06 ± 16.22	107.16 ± 14.69	100.80 ± 16.59	0.013*
FEV_1_ (L)	2.38 ± 0.79	2.55 ± 0.70	2.29 ± 0.82	0.079
%FEV_1_ (predicted, %)	90.98 ± 18.37	93.80 ± 15.93	89.41 ± 19.51	0.201
FEV_1_/FVC ratio (%)	73.46 ± 10.30	72.56 ± 8.29	73.97 ± 11.28	0.189
PEF (L/s)	7.23 ± 2.06	7.74 ± 1.94	6.95 ± 2.08	0.037*
%PEF (predicted, %)	103.28 ± 21.12	105.46 ± 19.00	102.07 ± 22.23	0.389
MMF (L)	1.95 ± 1.07	1.93 ± 0.95	1.95 ± 1.13	0.951
%MMF (predicted, %)	58.93 ± 27.49	57.98 ± 22.69	59.46 ± 29.95	0.773

Data are presented as the mean ± standard deviation unless otherwise indicated

Comparisons performed by Student’s t test, the Mann–Whitney U test, the Chi square test, and Fisher’s exact test as appropriate

*ACT* asthma control test, *AERD* aspirin-exacerbated respiratory disease, *BMI* body mass index, *FeNO* fractional exhaled nitric oxide, *FEV1* forced expiratory volume in 1 s, *FP* fluticasone propionate, *FVC* forced vital capacity, *GINA* Global Initiative for Asthma, *ICS* inhaled corticosteroid, *IgE* immunoglobulin E, *MMF* mid-maximal flow rate, *OCS* oral corticosteroids, *PEF* peak expiratory flow, *PSL* prednisolone, *Th2* T-helper cell type 2, *TNC* tenascin-C

*P < 0.05, GINA treatment steps 1–3 group versus GINA treatment steps 4 + 5

^†^Th2-high: total IgE level of more than 100 IU/mL and a peripheral blood eosinophil count of 0.14 × 10^9^ cells/L or more

### Association of serum periostin and TNC levels with subject characteristics in asthmatic patients

We next examined whether serum periostin and TNC levels in asthmatic patients were associated with subject characteristics. Serum periostin levels were positively correlated with age (r_s_ = 0.261, *P* = 0.003), age at asthma onset (r_s_ = 0.283, *P* = 0.001), ACT score (r_s_ = 0.24, *P* = 0.007), FeNO levels (r_s_ = 0.319, *P* < 0.001), peripheral blood eosinophil counts (r_s_ = 0.36, *P* < 0.001), and the Th2-high to Th2-low ratio (r_s_ = 0.195, *P* = 0.029) (Table 2). Although serum periostin levels were negatively correlated with the daily dose of inhaled corticosteroids (ICS) (r_s_ = − 0.194, *P* = 0.029) and the percentages of GINA treatment steps 4 + 5 (r_s_ = − 0.224, *P* = 0.012), periostin levels were also negatively correlated with airflow limitation, including FEV_1_ (r_s_ = − 0.203, *P* = 0.023), the mid-maximal flow rate (MMF) (r_s_ = − 0.25, *P* = 0.005), and percent predicted MMF (%MMF) (r_s_ = − 0.195, *P* = 0.028). Moreover, in the GINA step 4 + 5 group, serum periostin levels were positively correlated with age (r_s_ = 0.29, *P* = 0.009), age at asthma onset (r_s_ = 0.316, *P* = 0.004), FeNO levels (r_s_ = 0.226, *P* = 0.016), peripheral blood eosinophil counts (r_s_ = 0.398, *P* < 0.001), and the Th2-high to Th2-low ratio (r_s_ = 0.241, *P* = 0.03), but were negatively correlated with FVC (r_s_ = − 0.29, *P* = 0.009), FEV_1_ (r_s_ = − 0.295, *P* = 0.008) and MMF (r_s_ = − 0.286, *P* = 0.01) (Additional file 1: Table S1). Serum TNC levels were positively correlated with the percentages of GINA treatment steps 4 + 5 (r_s_ = 0.274, *P* = 0.002), daily dose of ICS (r_s_ = 0.206, *P* = 0.02), peripheral blood neutrophil counts (r_s_ = 0.189, *P* = 0.034), and serum total IgE levels (r_s_ = 0.259, *P* = 0.003) (Table 2). These results suggest that serum periostin and TNC levels were associated with distinct subject characteristics.

**Table 2:** Correlation coefficients for the association of serum periostin and TNC levels with subject characteristics in asthmatic patients

	Periostin		TNC	
	r_s_	*P* value	r_s_	*P* value
Sex (male)	− 0.033	0.711	− 0.025	0.780
Age (years)	0.261	0.003*	− 0.074	0.410
Age at asthma onset (years)	0.283	0.001*	− 0.019	0.831
Duration of asthma (years)	− 0.165	0.066	0.019	0.833
BMI (kg/m^2^)	− 0.071	0.428	0.029	0.749
Pack-year smoking history (pack year)	0.014	0.878	− 0.032	0.722
GINA step 4 + 5	− 0.224	0.012*	0.274	0.002*
AERD	0.119	0.186	0.132	0.142
Atopic dermatitis	− 0.016	0.857	0.003	0.969
Allergic rhinitis	− 0.041	0.651	− 0.027	0.763
Chronic sinusitis	0.088	0.329	0.026	0.771
Daily dose of ICS (FP equivalent dose, µg)	− 0.194	0.029*	0.206	0.020*
Daily dose of OCS (PSL equivalent dose, mg)	− 0.068	0.446	0.096	0.286
ACT score, n = 125	0.240	0.007*	− 0.108	0.232
FeNO (ppb)	0.319	< 0.001*	0.057	0.529
Peripheral neutrophils (cells/μL)	− 0.124	0.168	0.189	0.034*
Peripheral eosinophils (cells/μL)	0.360	< 0.001*	0.063	0.486
Serum IgE (IU/mL)	0.110	0.221	0.259	0.003*
Th2-high	0.195	0.029*	0.046	0.609
FVC (L)	− 0.167	0.061	− 0.056	0.536
%FVC (predicted, %)	− 0.047	0.604	− 0.104	0.246
FEV_1_ (L)	− 0.167	0.061	− 0.099	0.271
%FEV_1_ (predicted, %)	− 0.203	0.023*	− 0.042	0.642
FEV_1_/FVC ratio (%)	− 0.084	0.349	− 0.149	0.096
PEF (L/s)	− 0.146	0.103	− 0.057	0.524
%PEF (predicted, %)	− 0.024	0.786	− 0.103	0.252
MMF (L)	− 0.250	0.005*	− 0.058	0.522
%MMF (predicted, %)	− 0.195	0.028*	− 0.140	0.118

*ACT* asthma control test, *AERD* aspirin-exacerbated respiratory disease, *BMI* body mass index, *FeNO* fractional exhaled nitric oxide, *FEV1* forced expiratory volume in 1 s, *FP* fluticasone propionate, *FVC* forced vital capacity, *GINA* Global Initiative for Asthma, *ICS* inhaled corticosteroid, *IgE* immunoglobulin E, *MMF* mid-maximal flow rate, *OCS* oral corticosteroids, *PEF* peak expiratory flow, *PSL* prednisolone, *Th2* T-helper cell type 2, *TNC* tenascin-C

*P < 0.05

### Comparison of serum periostin and TNC levels between two subgroups according to asthma severity and Th2-related variables

**Fig. 1:**
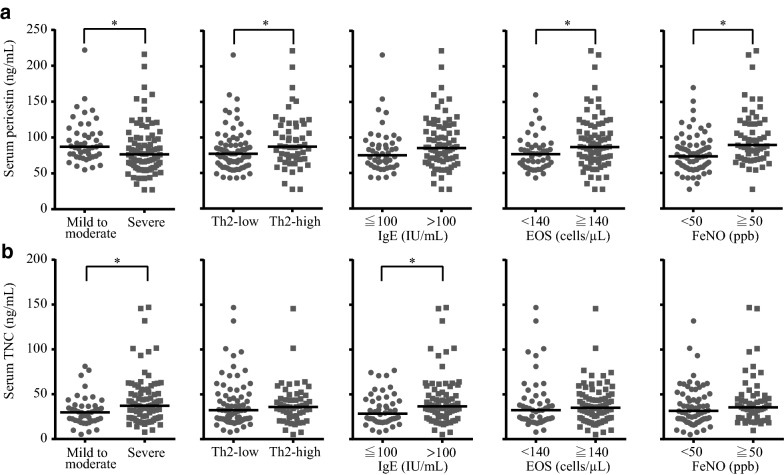
Association of serum periostin and TNC levels with asthma severity and T-helper cell type 2 (Th2)-related variables. **a** Serum periostin levels (ng/mL); and **b** serum TNC levels (ng/mL). Mild to moderate asthma was defined as well-controlled asthma requiring GINA treatment steps 1–3. Severe asthma was defined as asthma requiring GINA treatment steps 4/5 and as uncontrolled asthma despite the treatment. *P < 0.05, mild to moderate versus severe asthma, Th2-low versus Th2-high, serum immunoglobulin E (IgE) ≤ 100 versus IgE > 100, peripheral blood eosinophil count (EOS) < 140 versus EOS ≥ 140, fractional exhaled nitric oxide (FeNO) < 50 versus FeNO ≥ 50. Bars indicate median values

We then divided the 126 patients into two subgroups by five different ways: according to asthma severity (mild to moderate asthma and severe asthma), the Th2-high and Th2-low subgroups based on both serum IgE levels and a peripheral blood eosinophil counts, the high and low IgE subgroups based on serum IgE levels, the high and low eosinophil subgroups based on peripheral blood eosinophil counts, and the high and low FeNO subgroups based on FeNO levels (Fig. 1 and Additional file 2: Table S2). Serum periostin levels were significantly higher in patients with mild to moderate asthma (*P* = 0.01), Th2-high (*P* = 0.029), high peripheral blood eosinophil counts (≥ 0.14 × 10^9^ cells/L) (*P* = 0.01), and high FeNO levels (≥ 50 ppb) (*P* < 0.001) [Fig. 1a and Additional file 2: Table S2]. Serum TNC levels were significantly higher in patients with severe asthma (*P* = 0.012) and high serum total IgE levels (≥ 100 IU/mL) (*P* = 0.026) (Fig. 1b and Additional file 2: Table S2). These results suggest that serum periostin and TNC levels were associated with different characteristics of asthma disease severity and Th2-related variables. Moreover, not only serum periostin but also serum TNC might have potential use as novel biomarkers for asthma.

### Characteristics of patients with both high serum TNC levels and high serum periostin or IgE levels

**Fig. 2:**
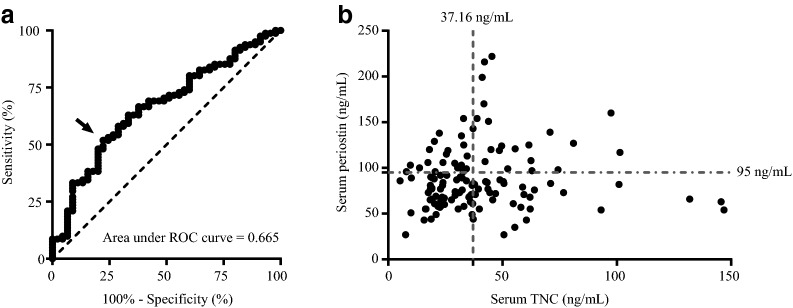
Relationship between serum TNC and periostin levels. **a** Receiver operating characteristic (ROC) curve for serum TNC levels comparing the GINA step 4 + 5 group with the GINA step 1–3 group. Using Youden’s index, the cut-off value for TNC of 37.16 ng/mL (sensitivity, 51.9%; specificity, 77.8%) is indicated with an arrow. **b** There was no correlation between serum periostin and TNC levels (r_s_ = 0.111, *P* = 0.216). Asthmatic patients were divided into four groups according to the cut-off values for serum TNC levels (37.16 ng/mL) and serum periostin levels (95 ng/mL)

We evaluated whether the combination of serum periostin and TNC levels were more reliable than a single biomarker approach. Receiver operating characteristic curve analysis was used to determine the optimal cut-off value of serum TNC level to discriminate the GINA step 4 + 5 group from the GINA step 1–3 group, with the area under the curve of 0.665 (95% CI 0.57–0.76) (Fig. 2a). A serum TNC level of 37.16 ng/mL was the best cut-off value for the optimal potential effectiveness of serum TNC using Youden’s index [37]. There were no correlation between serum periostin and TNC levels (r_s_ = 0.111, *P* = 0.216) (Fig. 2b). We then divided the 126 patients into four subgroups according to the cut-off values for serum TNC (37.16 ng/mL) and serum periostin (95 ng/mL) (Fig. 2b, Table 3 and Additional file 3: Table S3) [19]. In patients with high serum TNC and periostin levels, the percentages of GINA treatment steps 4 + 5 (*P* = 0.042), percentages of patients with aspirin-exacerbated respiratory disease (AERD) (*P* = 0.004), daily dose of ICS (*P* = 0.045), and peripheral blood eosinophil (*P* = 0.005) and neutrophil counts (*P* = 0.032) were significantly higher, whereas FVC (*P* = 0.01), %FVC (*P* = 0.019), FEV_1_ (*P* = 0.014), PEF (*P* = 0.045), MMF (*P* = 0.045), and %MMF (*P* = 0.042) were significantly lower as compared with patients in the other subpopulations (Table 3). These data suggest that the combination of serum periostin and TNC had the ability to reflect asthma severity and airflow limitation in asthmatic patients.

**Table 3:** Characteristics that are statistically different between patients with high serum TNC and high serum periostin levels and others

	High TNC > 37.16	Low TNC < 37.16	High TNC > 37.16	Low TNC < 37.16	*P* value for multigroup analysis^†^	Groups B, C and D	*P* value between groups A and E^‡^
	High periostin > 95	High periostin > 95	Low periostin < 95	Low periostin < 95			
	A (n = 20)	B (n = 21)	C (n = 32)	D (n = 53)		E (n = 106)	
GINA step 4 + 5, n (%)	17 (85.0)	7 (33.3)	25 (78.1)	32 (60.4)	0.001*	64 (60.4)	0.042*
AERD, n (%)	6 (30.0)	0 (0.0)	1 (3.1)	5 (9.4)	0.004*	6 (5.7)	0.004*
Daily dose of ICS (FP equivalent dose, µg)	735.00 ± 346.83	392.86 ± 346.51	629.69 ± 349.39	575.47 ± 407.10	0.021*	555.66 ± 384.91	0.045*
FeNO (ppb)	65.30 ± 46.36	65.87 ± 34.04	54.51 ± 52.86	47.21 ± 39.25	0.033*	53.11 ± 43.12	0.208
Peripheral neutrophils (cells/μL)	4588.06 ± 1452.31	3496.31 ± 1052.69	4216.49 ± 1600.66	3900.12 ± 1535.39	0.037*	3915.63 ± 1482.52	0.032*
Peripheral eosinophils (cells/μL)	423.03 ± 315.58	354.16 ± 243.56	223.62 ± 224.07	191.55 ± 159.50	0.001*	233.44 ± 206.28	0.005*
Serum periostin (ng/mL)	138.65 ± 37.94	114.33 ± 17.18	67.81 ± 15.66	69.81 ± 14.82	< 0.001*	78.03 ± 23.81	< 0.001*
Serum TNC (ng/mL)	57.93 ± 18.62	25.42 ± 8.56	62.44 ± 29.95	24.25 ± 7.94	< 0.001*	36.01 ± 24.80	< 0.001*
FVC (L)	2.72 ± 0.93	3.17 ± 0.86	3.38 ± 0.89	3.34 ± 0.98	0.063	3.32 ± 0.93	0.010*
%FVC (predicted, %)	96.10 ± 9.38	103.77 ± 16.30	102.95 ± 16.61	105.47 ± 17.57	0.118	104.37 ± 16.92	0.019*
FEV_1_ (L)	1.99 ± 0.80	2.28 ± 0.59	2.47 ± 0.83	2.52 ± 0.80	0.062	2.46 ± 0.77	0.014*
PEF (L/s)	6.39 ± 2.00	6.92 ± 1.42	7.46 ± 2.35	7.54 ± 2.05	0.142	7.39 ± 2.04	0.045*
MMF (L)	1.51 ± 1.01	1.64 ± 0.69	1.99 ± 1.07	2.21 ± 1.14	NS	2.03 ± 1.06	0.045*
%MMF (predicted, %)	48.14 ± 24.36	54.37 ± 20.36	56.33 ± 26.21	66.39 ± 30.28	NS	60.97 ± 27.67	0.042*

Data are presented as the mean ± standard deviation unless otherwise indicated

*ACT* asthma control test, *AERD* aspirin-exacerbated respiratory disease, *BMI* body mass index, *FeNO* fractional exhaled nitric oxide, *FEV1* forced expiratory volume in 1 s, *FP* fluticasone propionate, *FVC* forced vital capacity, *GINA* Global Initiative for Asthma, *ICS* inhaled corticosteroid, *IgE* immunoglobulin E, *MMF* mid-maximal flow rate, *OCS* oral corticosteroids, *PEF* peak expiratory flow, *PSL* prednisolone, *Th2* T-helper cell type 2, *TNC* tenascin-C, *NS* not significant

*P < 0.05

^†^Multigroup analysis performed by Chi square test, One-way ANOVA and Kruskal–Wallis test as appropriate

^‡^Comparisons performed by Student’s t test, the Mann–Whitney U test, and Fisher’s exact test as appropriate

We next evaluated whether the combination of serum TNC and total IgE levels were more reliable than a single biomarker approach, as described above for periostin. We also divided the 126 patients into four subgroups according to the cut-off values for serum TNC (37.16 ng/mL) and serum total IgE levels (100 IU/mL) (Table 4 and Additional file 4: Table S4). The percentages of GINA treatment steps 4 + 5 (*P* = 0.023), percentages of patients with Th2-high (*P* = 0.003), and peripheral blood neutrophil counts (*P* = 0.002) were significantly higher, whereas %FVC (*P* = 0.005), %FEV_1_ (*P* < 0.001), percent predicted PEF (*P* = 0.033), and %MMF (*P* = 0.01) were significantly lower in patients with high serum TNC and total IgE levels as compared with patients in the other subpopulations (Table 4). These data suggest that the combination of serum TNC and IgE also had the ability to reflect asthma severity and airflow limitation in asthmatic patients.

**Table 4:** Characteristics that are statistically different between patients with high serum TNC and high serum IgE levels and others

	High TNC > 37.16	Low TNC < 37.16	High TNC > 37.16	Low TNC < 37.16	*P* value for multigroup analysis^†^	Groups B, C and D	*P* value between groups A and E^‡^
	High IgE > 100	High IgE > 100	Low IgE < 100	Low IgE < 100			
	A (n = 36)	B (n = 42)	C (n = 16)	D (n = 32)		E (n = 90)	
GINA step 4 + 5, n (%)	29 (80.6)	18 (42.9)	13 (81.3)	21 (65.6)	0.002*	52 (57.8)	0.023*
Peripheral neutrophils (cells/μL)	4593.71 ± 1656.42	3823.77 ± 1249.02	3832.20 ± 1117.89	3735.33 ± 1637.25	0.018*	3793.82 ± 1365.61	0.002*
Peripheral eosinophils (cells/μL)	338.37 ± 302.52	287.01 ± 206.42	214.70 ± 194.18	172.96 ± 172.63	0.027*	233.60 ± 197.73	0.170
Serum IgE (IU/mL)	1120.92 ± 2424.39	831.76 ± 1733.44	46.26 ± 28.61	51.09 ± 25.59	< 0.001*	414.55 ± 1240.42	< 0.001*
Th2-high, n (%)	23 (63.9)	30 (71.4)	0 (0.0)	0 (0.0)	< 0.001*	30 (33.3)	0.003*
Serum TNC (ng/mL)	63.77 ± 29.77	26.04 ± 8.42	53.79 ± 12.95	22.65 ± 7.29	< 0.001*	29.77 ± 14.42	< 0.001*
%FVC (predicted, %)	97.08 ± 12.80	105.44 ± 16.51	107.59 ± 16.01	104.40 ± 18.16	NS	105.45 ± 16.88	0.005*
%FEV_1_ (predicted, %)	82.77 ± 15.57	94.92 ± 16.70	95.69 ± 19.99	92.69 ± 20.27	0.005*	94.26 ± 18.45	< 0.001*
%PEF (predicted, %)	96.95 ± 19.02	103.93 ± 19.00	107.31 ± 24.44	107.53 ± 23.42	0.124	105.81 ± 21.48	0.033*
%MMF (predicted, %)	48.56 ± 20.87	63.05 ± 24.47	63.56 ± 32.29	62.88 ± 32.92	0.083	63.08 ± 28.79	0.010*

Data are presented as the mean ± standard deviation unless otherwise indicated

*ACT* asthma control test, *AERD* aspirin-exacerbated respiratory disease, *BMI* body mass index, *FeNO* fractional exhaled nitric oxide, *FEV1* forced expiratory volume in 1 s, *FP* fluticasone propionate, *FVC* forced vital capacity, *GINA* Global Initiative for Asthma, *ICS* inhaled corticosteroid, *IgE* immunoglobulin E, *MMF* mid-maximal flow rate, *OCS* oral corticosteroids, *PEF* peak expiratory flow, *PSL* prednisolone, *Th2* T-helper cell type 2, *TNC* tenascin-C, *NS* not significant

*P < 0.05

†Multigroup analysis performed by Chi square test, One-way ANOVA and Kruskal–Wallis test as appropriate

‡Comparisons performed by Student’s t test, the Mann–Whitney U test, and Fisher’s exact test as appropriate

### Serum TNC levels and the therapeutic effect of omalizumab for patients with severe asthma

Twenty-one (16.7%) asthmatic patients had been treated with omalizumab, a recombinant humanized anti-IgE monoclonal antibody for severe asthma, prior to enrolling in this study. Serum TNC levels in omalizumab-treated patients were significantly higher than those in patients not treated with omalizumab (52.72 ± 31.71 ng/mL versus 36.84 ± 22.94 ng/mL; *P* = 0.014), which corresponded to previously shown results that serum TNC levels were correlated with asthma severity and the daily dose of ICS. The mean duration of omalizumab treatment and median age were 26.89 ± 17.15 months (range 0.93–66.23 months) and 53 years (range 20–86 years), respectively.

**Fig. 3:**
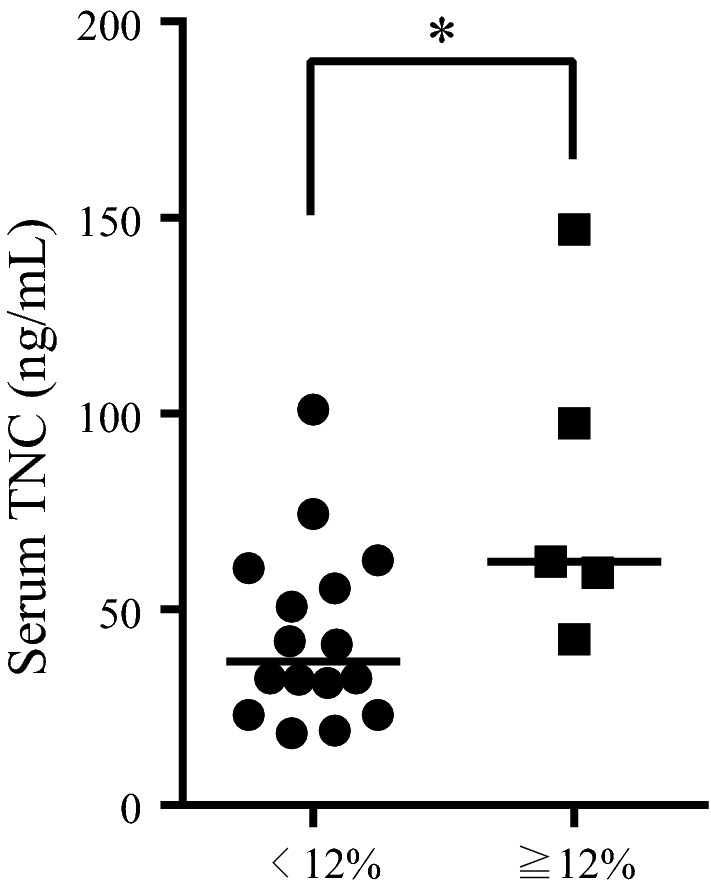
Serum TNC levels and the therapeutic effect of omalizumab for patients with severe asthma. Serum TNC levels were significantly higher in the subgroup with an improvement in forced expiratory volume in 1 s (FEV_1_) of ≥ 12% than that in the subgroup with improvement in FEV_1_ of < 12%. *P < 0.05

Finally, we investigated whether serum TNC levels were associated with the effect of omalizumab treatment. The 21 patients were divided into two subgroups according to change in FEV_1_ of more or less than 12% of baseline, i.e., the ratio of FEV_1_ at enrollment after treatment to baseline FEV_1_ before treatment. Only serum TNC levels showed a significant difference between the two subgroups among evaluated subject characteristics (Additional file 5: Table S5). Serum TNC levels were significantly higher in the subgroup with an improvement in FEV_1_ of ≥ 12% than that in the subgroup with improvement in FEV_1_ of < 12% (Fig. 3). Moreover, all of the patients with an improvement in FEV_1_ of ≥ 12% were included in the subgroup with high serum TNC levels (> 37.16 ng/mL) and serum periostin was not associated with the omalizumab-related improvement subgroup (Additional file 6: Table S6 and data not shown).

## Discussion

The results of the present study confirm previous reports by showing that serum TNC concentrations in patients with asthma were associated with disease severity [29]. Furthermore, to our knowledge, this is the first study to show that serum TNC levels in asthmatic patients are associated with clinical features of asthma and that using both the combination of serum TNC and periostin levels and the combination of serum TNC and total IgE levels in a multiple-marker approach might be a more useful biomarker for asthma. The present study demonstrated that peripheral blood eosinophil counts and total serum IgE levels were associated with serum periostin and TNC levels, respectively. Moreover, disease severity, percentages of patients with AERD, and airflow limitation were associated with patients with high serum TNC and periostin levels as compared with patients in the other subpopulations, suggesting that both periostin and TNC might serve as biomarkers of asthma. It was reported that the gene expression of periostin and TNC in bronchial epithelial cells is upregulated by Th2 cytokines, including IL-4 and IL-13, and that the secretion of periostin and TNC in lung fibroblasts is also induced by both IL-4 and IL-13 [6, 10, 11]. Both periostin and TNC bind to each other and also co-localize in subepithelial fibrosis in asthmatic patients [6]. Although the production of both extracellular matrix proteins is induced by IL-4 and IL-13, it is interesting to note that different features were observed between serum periostin and TNC levels in asthmatic patients in the present study. IgE synthesis is also regulated by IL-4 and IL-13 [38, 39]. Previous report demonstrates that IgE in the bronchoalveolar lavage fluid are significantly decreased in ovalbumin-induced asthma mice model using TNC-deficient mice and that addition of exogenous TNC to mouse spleen lymphocytes stimulates IgE secretion [28]. These data suggests that TNC has a potential of IgE synthesis. On the other hand, there are two reports using different periostin-deficient mice. One report shows that allergen-induced increases in serum IgE and airways hyperresponsiveness are exaggerated in periostin-deficient mice challenged with inhaled *Aspergillus fumigatus* antigen [40]. Another report using periostin-deficient mice and anti-periostin neutralizing antibody shows that periostin is required for IgE synthesis and airways hyperresponsiveness in mice challenged with inhaled aeroallergen, house dust mite [41]. These results suggest that periostin and TNC may have different function for IgE synthesis and may reflect their different features. Because both serum periostin and TNC levels were not correlation and had different features, the combination of serum TNC and periostin levels in a multiple-marker approach might be more useful biomarkers reflecting asthma severity including airflow limitation than a single biomarker approach.

TNC is a matricellular protein that is highly expressed during wound healing and tissue remodeling processes in chronic inflammation, including asthma [24, 42–44]. The results of the present study demonstrated that serum TNC levels were not correlated with airflow limitation despite a correlation with asthma severity and high serum IgE levels, even when the asthma was severe. However, this study suggested that serum TNC levels may reflect disease severity in asthma and may be an indicator of airflow limitation in asthmatic patients with high serum periostin levels or high serum total IgE levels. Moreover, serum TNC levels were associated with peripheral blood neutrophil counts in the especially periostin-high subgroup or IgE-high subgroup, suggesting that serum TNC levels may reflect not only type 2 airway inflammation but also neutrophilic airway inflammation.

High serum TNC levels have already some application as biomarker. Increased levels of serum TNC might be useful in liver fibrosis [45], inflammatory bowel diseases [46, 47], cardiovascular diseases [48–51], and refractory asthma [29]. Serum TNC levels in patients with inflammatory bowel disease correlate with disease severity [46], and infliximab therapy response in patients with ulcerative colitis is associated with decreased levels of serum TNC [47]. In patients with dilated cardiomyopathy, high serum TNC levels might indicate the severity of heart failure, left ventricular (LV) dysfunction and remodeling [48–50]. Moreover, previous report on acute myocardial infarction (AMI) shows that serum TNC levels in patients with AMI is significantly elevated, peaks at day 5, and then gradually decreases, and suggests that serum TNC levels might be useful in predicting LV remodeling and prognosis after AMI [51]. These applications and the results of present study suggests that serum TNC might be a novel marker reflecting active structural remodeling in fibrosis, inflammatory bowel diseases, cardiovascular diseases, and asthma.

In previous studies, the serum periostin level had potential as a single biomarker to predict eosinophilic airway inflammation and risk of a decline in FEV_1_ in asthmatic patients and was associated with late onset, high eosinophil counts, AERD, and chronic sinusitis [15–17, 19–21, 52]. Our results confirmed that high serum periostin levels were correlated with late onset and high peripheral blood eosinophil counts, but demonstrated that high serum periostin levels were not correlated with AERD and chronic sinusitis. However, the percentages of patients with AERD among patients with high serum TNC and periostin levels were higher than those in other subgroups, and serum periostin levels were correlated with AERD in the high serum TNC subgroup (data not shown). Furthermore, previous reports suggested that high serum periostin is associated with asthma severity [17, 18]. The present study showed that serum periostin levels were correlated with airflow limitation and showed a better correlation with airflow limitation in patients with severe asthma and high serum TNC levels. However, the present study also showed an inverse correlation between serum periostin and asthma disease severity, i.e., patients with mild to moderate asthma had high serum periostin levels. The reason for the discrepancy between serum periostin levels and asthma severity was not clear in the current study. This discrepancy may be related to dominant low FeNO levels in patients with severe asthma. Nevertheless, the findings of this unique subpopulation may lead to discrepant results between previous studies and the current study.

Omalizumab, a recombinant humanized monoclonal antibody against human IgE, has important benefits as an add-on therapy for patients with inadequately controlled severe persistent asthma who have a significant unmet need [53–56]. However, not all patients with inadequately controlled asthma respond to omalizumab and predictors of response to this biological therapy are limited [57]. It has been reported that serum IgE levels and antigen-specific IgE could not predict the response to omalizumab [58–60]. The EXTRA omalizumab study suggested the potential of three biomarkers of Th2-driven inflammation, including FeNO levels, peripheral blood eosinophil counts, and serum periostin levels, as predictors of the response to omalizumab to reduce the incidence of severe exacerbation [57]. In the present study, we demonstrated that the omalizumab-related improvement in FEV_1_ of at least 12% was associated with high serum TNC levels, indicating that patients with high serum TNC levels may achieve a greater benefit from omalizumab therapy.

There were several important limitations to this study. First, the lack of data of healthy subjects is a limitation. Second, for data on omalizumab treatment (Additional file 5: Table S5), serum TNC levels were evaluated after approximately 2 years from starting omalizumab treatment, blood samples were collected at different time points after starting treatment, and the sample size was small. Therefore, the data for omalizumab treatment should be considered preliminary. Further studies are needed to investigate whether serum TNC levels and/or the combination of serum TNC and periostin levels can serve as more useful biomarkers in asthmatic patients and whether it has the potential as a biomarker to predict the therapeutic efficacy of omalizumab for severe asthmatic patients.

## Conclusions

We have provided the first report that serum TNC levels in asthmatic patients were associated with clinical features of asthma and that the combination of serum TNC and periostin levels or the combination of serum TNC and total IgE levels were more useful for asthma than a single biomarker approach, suggesting that serum TNC can serve as a novel biomarker for asthma. Additional studies are needed to investigate whether serum TNC levels and/or combination with other markers are more useful biomarkers in asthmatic patients.

## Additional files

**Additional file 1: Table S1.** Correlation coefficients for the association of serum periostin and TNC levels with subject characteristics, stratified by GINA steps.

**Additional file 2: Table S2.** The statistical significance and confidence intervals for serum periostin and TNC levels with asthma severity and Th2-related variables.

**Additional file 3: Table S3.** Characteristics of patients divided into four groups according to serum TNC and periostin levels.

**Additional file 4: Table S4.** Characteristics of patients divided into four groups according to serum TNC and IgE levels.

**Additional file 5: Table S5.** Characteristics of omalizumab-treated patients.

**Additional file 6: Table S6.** Omalizumab-treated patients to divide into 4 groups according to serum TNC and Periostin levels.

## Abbreviations

ACT: asthma control test
AERD: aspirin-exacerbated respiratory disease
BMI: body mass index
ELISA: enzyme-linked immunosorbent assay
FeNO: fractional exhaled nitric oxide
FEV_1_: forced expiratory volume in 1 s
FP: fluticasone propionate
FVC: forced vital capacity
GINA: Global Initiative for Asthma
ICS: inhaled corticosteroid
IgE: immunoglobulin E
IL: interleukin
MMF: mid-maximal flow rate
OCS: oral corticosteroids
PEF: peak expiratory flow
PSL: prednisolone
Th2: T-helper cell type 2
TNC: tenascin-C
